# Early Pregnancy Loss Following Laparoscopic Management of Ovarian Abscess Secondary to Oocyte Retrieval

**Published:** 2014-11-01

**Authors:** Emre Goksan Pabuccu, Salih Taskin, Cem Atabekoglu, Murat Sonmezer

**Affiliations:** 1 Department of Obstetrics and Gynecology, Ankara University School of Medicine, Ankara, Turkey; 2 Division of Reproductive Medicine and Gynecologic Endocrinology, Ankara University School of Medicine, Ankara, Turkey

**Keywords:** Abscess, Laparoscopy, Oocyte Retrieval, Pelvic Infection

## Abstract

Severe pelvic infections following ultrasound-guided transvaginal oocyte retrieval (TVOR) are rare but challenging. Ovarian abscess formation is one of the consequences and management of such cases as highly debated in pregnant patients. In this case report, an early fetal loss following laparoscopic management of ovarian abscess is described and possible etiologies are discussed.

## Introduction

Pelvic infections following ultrasound-guided transvaginal oocyte retrieval (TVOR) are rare complications with an incidence of 0.6% (1). The procedure is generally considered safe; however, vaginal flora acts as the main reservoir for microorganisms and bacterial inoculation via retrieval needle is possible.. Pelvic abscess formation is very rare, and until now, 8 cases have been reported in the literature (2-9). There is no standard approach available and management becomes more complicated, especially in pregnant patients. Pelvic infections could be a potential threat to an early pregnancy, and hence an early diagnosis and a thorough management are of paramount importance both for the patient and the ongoing pregnancy. This case report intends to discuss different treatment strategies and to question their reliabilities, especially during early pregnancy period.

## Case Report

A 26-year-old, nulliparous woman underwent in vitro fertilization (IVF) for male factor subfertility in an assisted reproduction unit, Ankara, Turkey. Her medical history was unremarkable and physical examination was normal. Basal ultrasound examination on the 3rd day of menstrual cycle revealed a normal pelvic anatomy without appearance of an ovarian cyst including endometrioma. Following a standard ovarian stimulation with gonadotropins, TVOR was performed at the 36^th^ hour of ovulation trigger using 250 µg of recombinant human chorionic gonadotropin (rhCG, ovitrelle, Merck Serono, Turkey). Retrieval process was uneventful without any complications. The patient was prescribed a 5-day course of oral doxycycline (Monodoks, Deva, Turkey) (100 mg, twice daily) as a part of routine medication after retrieval process and vaginal micronized progesterone (Progestan, Kocak, Turkey) (200 mg, three times a day) for luteal phase support. On the 14^th^ day of transfer, pregnancy was confirmed with a quantitative betahCG (ß-hCG) value of 240 mIU/ml. A subsequent doubling in serum hCG levels was also observed suggestive of an early ongoing intrauterine pregnancy. Three weeks after the retrieval procedure, the patient was admitted to the emergency unit with mild abdominal pain and elevated body temperature (38.3℃). Her physical examination revealed rebound tenderness in the lower abdomen and tenderness during bimanual pelvic examination. Pelvic sonography revealed a 4.5×4 cm echogenic cystic mass on the left adnexa with mild fluid in the Douglas pouch. Laboratory tests were within the normal range except for leukocytosis (12.500/mm^3^) and elevated C-reactive protein (8 mg/L) (CRP). An ultrasound-guided drainage of the mass was considered, but due to the anatomic position of the mass and poor cooperation from the patient, the procedure was not successful. Thus an initial empirical antibiotic therapy was started within travenous (IV) ceftriaxone sodium (Iesef, Ulagay, Turkey) 1gr twice a day and metronidazole 500 mg (Flagyl, Sanofi Aventis, Turkey) three times a day, as we were also unable to obtain satisfactory amount of culture sample from Douglas pouch. Despite full course of antibiotics for 48 hours, mild fever (38℃) and abdominal tenderness persisted and a laparoscopic drainage was decided. On initial evaluation, formations of left ovarian abscess and diffuse pus were confirmed (Figes1, 2). Drainage and excision of the abscess wall were performed and whole pelvis was irrigated with 3 liters of saline (Fig 3). Antibiotics and progesterone supplementation were continued following laparoscopy. Following an uneventful recovery, the patient was discharged five days after the surgery. Escherichia coli, bacteroides and peptostreptococcus species were reported to be isolated in the abscess culture. A dichorionic-diamniotic twin pregnancy with cardiac activities was confirmed during her routine obstetric follow-up at the 5th week of gestation. However, three weeks later, unfortunately ultrasound investigation failed to confirm both cardiac activities. There were no signs and symptoms suggestive of either persisting or recurrence of infection in the patient at that stage. An informed consent was obtained from patient before submitting the case into the journal.

**Fig 1:**
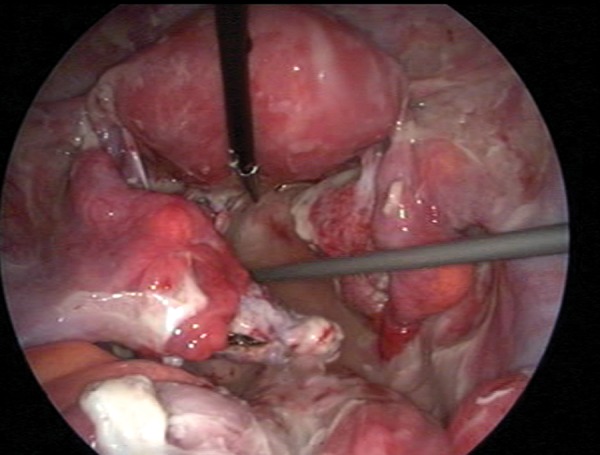
The initial view of the pelvis, depicting a left ovarian mass consistent with abscess, disseminated purulent fluid.

**Fig 2:**
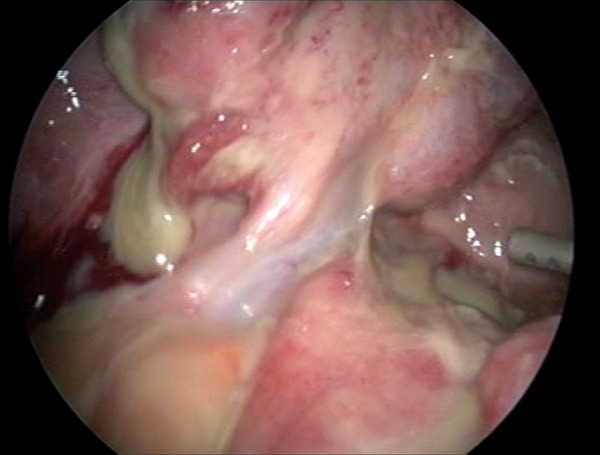
Drainage of purulent fluid from the left ovarian mass.

**Fig 3:**
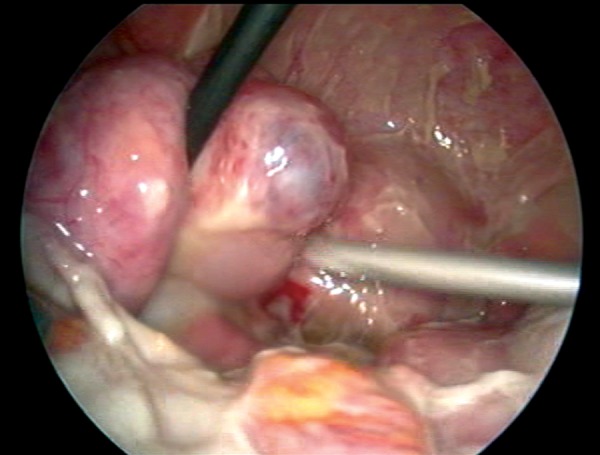
Appearance after excision of the mass and irrigation of the pelvis.

## Discussion

Oocyte retrieval process is an invasive procedure with the potential risk of inoculation of the vaginal microorganisms into abdomen. The risk of pelvic infection after TVOR is estimated as 0.6% (1). It may be caused not only by inoculation of vaginal microorganisms, but also by reactivation of a latent pelvic inflammation or direct intestinal injury (4). The interval between the procedure and occurrence of symptoms is variable. According to the literature (Table 1), this was reported as shorter than 25 days in almost half of the patients; however, prolonged intervals have also been reported (3, 4). This interval might differ according to the virulence of microorganisms or immune response of the patient. Use of prophylactic antibiotics following oocyte retrieval is controversial as pelvic inflammation is uncommon and these medications may not prevent all associated infections. Hence, antibiotics should be considered for at-risk patients, such as those with endometriosis or history of pelvic infection or surgery (1, 3). On the other hand, presence of vaginal-cervical microbial contamination at the time of embryo transfer is associated with significantly decreased pregnancy rates (10). Therefore, empirical antibiotic decision mostly correlates with clinician’s experience and opinion. In our case, none of the above-mentioned risk factors were present; however, a prophylactic doxycycline was prescribed due to relatively high prevalence of chlamydial infections among reproductive age patients in our population.

Topical antiseptic usage for vaginal preparation before TVOR is another controversial issue in the presence of risk factors. However, there has been no such accepted universal approach. Povidone-iodine or chlorhexidine are commonly used to sterilize the vagina, whereas other options include saline irrigation, careful removal with dry swabs, or avoiding them completely by cleansing the vagina with only saline solutions. Tsai et al. (11) reported that vaginal douching with the addition of aqueous povidone-iodine is effective in preventing the infection without compromising the outcome of the IVF treatment. In the present case, povidone-iodine and further vaginal irrigation with saline was performed.

**Table 1:** Review of cases with Pelvic abscess formation following oocyte retrieval in the literature


Reference	Age (Y)	Opu procedure	Time of symptoms	Possible risk factor(s)	Treatment	Maternal / fetal outcome

**Biringer et.al.(2)**	32	NA	16^th^ week of gestation	Unilateral salpingectomy for ectopic pregnancy	Antibiotherapy, delivery of a first fetus at 16^th^ week and laparotomic drainage	Delivery of second fetus without complications at 30^th^ week
**Den Boon et.al.(3)**	36	NA	25 week 4 days of gestation	Surgery for endometriosis and presence of endometrioma during OPU	Laparotomic bilateral multiple ovarian abscess drainage and antibiotherapy.	Delivery at 26 weeks, hypoalbuminemia , pulmonary edema, re-laparotomy for peritonitis and ileus 1. Baby: After treatment for prematurity related complications, he is well and 8 months old; 2. Baby: died at 9th week with severe brain damage
**Sharpe et.al.(4)**	35	Vagina cleansed with saline	13^th^ week of gestation	Endometriosis and aspiration of an endometrioma during OPU	Antibiotherapy and observation	Delivery at 31^st^ week by C-section. Drain left to pelvis and abscess resolved completelyThere were no neonatal complications
**Matsunaga et.al.(5)**	35	NA	16^th^ week of gestation	Presence of endometriosis and endometrioma	Antibiotherapy at 16^th^ and 20th week. Delivery of unviable fetus at 22nd week and laparotomic left salpingoophorectomy for large abscess	Unevet Full postoperative course
**Younis et.al.(6)**	29	NA	22 days after oocyte pick up	Bilaterally endometriomas	Antibiotherapy without surgical intervention	Delivery at term without neonatal or maternal complications
**Padilla et.al.(7)**	34	Vaginal iodinization followed by saline irrigation	21 days after oocyte pick up	Aspiration of endometrioma during OPU	Antibiotherapy and L/S drainage of abscess	7 weeks of ongoing pregnancy
**Jahan and Powell (8)**	27	NA	23^rd^ day of IVF cycle	Presence of endometrioma	Antibiotherapy and L/S drainage (interval of 5 days between 2 L/S)	Delivery at 37^th^ week of gestation without any maternal complications. Newborn was operated for cardiac anomaly and well after the operation
**Zweemer et al. (9)**	34	NA	36^th^ week of gestation	Surgery for tubal pregnancy	NA	Delivery at 38^th^ week of gestation without any maternal complications
**Present case**	26	Vaginal iodinization followed by saline irrigation	21 days after OPU	No	Antibiotherapy and L/S drainage of abscess	Missed abortus at 8^th^ week of gestation


NA; Not available, OPU; Oocyte pick up, L/S;Laparoscopy and IVF; In vitro fertilization.

Life threatening complications as a result of assisted reproductive techniques obviously require close surveillance and active management. Ideally, less invasive and conservative approaches should be the first option; nevertheless, severe complications such as pelvic abscess sometimes require more aggressive treatment. Trans-vaginal ultrasound-guided drainage of pelvic abscess is a relatively easy, safe and effective procedure. It has also been proven to be significantly more effective than medical therapy and has been associated with a low surgical morbidity (12). It is, therefore, suggested as the first-line procedure for the treatment of tubal-ovarian abscess. In this case, drainage was considered unsafe due to anatomic malposition of the mass, as it was visualized just behind the corpus uteri in vaginal sonography. As conservative medical management was not successful, laparoscopic surgery was decided and successful excision of the abscess wall along with whole abdomen irrigation was performed. For non-urgent conditions, the second trimester of pregnancy has classically been considered as the safest period for surgical intervention. Utero-placental oxygenation, adequate fetal perfusion and avoidance of teratogenic drugs are essential factors to be considered when embarking on an endoscopic surgery in a pregnant patient (13, 14). During the procedure, physiologic outcomes of pneumo-peritoneum to the fetus must be carefully considered, since carbon-dioxide (CO_2_) is highly diffusible and may induce fetal tachycardia and acidosis (15). Amos et al. (16) reported four fetal deaths in seven pregnant women who underwent laparoscopic cholecystectomy or appendectomy, and authors also suggested that fetal demise could have been due to prolonged respiratory acidosis, despite maintaining end-tidal carbon dioxide (EtCO_2_) in the physiologic range. On the other hand, Steinbrook and Bhavani-Shankar (17) reported a case series of ten pregnant women, with gestational ages of 9 to 30 weeks undergoing laparoscopic cholecystectomy and no adverse maternal or fetal outcomes were noted. Additionally, a recent guideline has already showed that laparoscopy can safely be performed during any trimester of pregnancy and has no disadvantage compared to laparotomy (18).

Management of tubal-ovarian abscess is quite complicated in women of reproductive age, and especially in pregnant patients. Today, in the light of growing evidence, majority of clinicians choose to perform fertility-sparing procedures in management of pelvic abscess. Laparoscopy provides direct visualization of pelvis and allows clinicians to perform additional procedures such as adhesiolysis, salpingotomy, and excision of necrotic tissues, simultaneously.

Even though the exact cause of fetal loss is unclear in this case report, pelvic infection can be assumed to be the plausible cause as there was evidence of inflammation reported in the pathological examination of the fetal material. As the duration between surgery and fetal loss was relatively long, it is really hard to say that fetal loss may be linked with the surgical procedure.Long duration of parenteral antibiotic usage is another questionable issue in this case for its toxic consequences on the fetus. However, beyond the etiology of the loss, this report was aimed at raising the issue of alternative management of an abscess when the case is not suitable for ultrasound-guided drainage. In seeking for a more accurate and safe method, especially applicable in early pregnancy, further studies are definitely required.

## Conclusion

In sum, formation of pelvic abscess following TVOR is a rare, but a serious complication and laparoscopy may be a feasible option when less invasive approaches are unsuccessful during early pregnancy.
